# Prelacteal feeding practices and associated factors among mothers of children aged less than 24 months in Raya Kobo district, North Eastern Ethiopia: a cross-sectional study

**DOI:** 10.1186/s13006-014-0025-2

**Published:** 2014-12-14

**Authors:** Misgan Legesse, Melake Demena, Firehiwot Mesfin, Demewoz Haile

**Affiliations:** Department of Nursing, College of Medicine and Health Sciences, Samara University, Samara, Ethiopia; Department of Public Health, College of Health Science and Medicine, Haramaya University, Harar, Ethiopia; Department of Public Health, College of Medicine and Health Sciences, Madawalabu University, Bale Goba, Ethiopia

**Keywords:** Prelacteal, Children aged less than 24 months, Ethiopia

## Abstract

**Background:**

The harmful infant feeding practices of prelacteal feeding is widely practiced in Ethiopia. Hence, it is vital to appreciate the cultural basis and potential factors on infant feeding practices in different parts of Ethiopia. This study aimed to investigate prelacteal feeding practices and associated factors among mothers of children aged less than 24 months in Raya Kobo district, North Eastern Ethiopia.

**Methods:**

A quantitative community-based cross-sectional study supplemented by qualitative methods was employed. Sixty hundred thirty (630) mothers of children aged less than 24 months were selected by systematic random sampling technique. Descriptive statistics, bivariate and multivariable logistic regression analysis were employed to identify the factors associated with prelacteal feeding practices. Variables with a p-value < 0.05 were identified as statistically significant factors. Qualitative data was collected by focus group discussion and in-depth interview and analyzed using thematic frameworks.

**Results:**

The prevalence of prelacteal feeding was 38.8% (95% CI: 35.00%, 43.00%). Home delivery was a risk factor for practicing prelacteal feeding. Those mothers who gave birth at home were seven times more likely to practice prelacteal feeding as compared to mothers who delivered at health institutions (Adjusted Odd Ratio (AOR):7.10; 95% CI: 3.91, 12.98). Mothers who were not aware of the risks associated with prelacteal feeding were nearly four times more likely to practice prelacteal feeding as compared to knowledgeable mothers (AOR: 3.70; 95% CI: 2.44, 5.53). Late initiation of breastfeeding (after one hour of delivery) was also associated with prelacteal feeding practice (AOR: 2.70; 95% CI: 1.78, 3.99). The major reasons stated for providing prelacteal feeding were to prevent “evil eye” and illness and to “clean infant’s stomach”.

**Conclusion:**

Prelacteal feeding was commonly practiced in Raya Kobo district. Home delivery, delayed commencement of breastfeeding after birth and lack of awareness about the risks associated with prelacteal feeding were predictors of prelacteal feeding. Therefore, strengthening infant feeding counseling about the risks associated with prelacteal feeding, promoting institutional delivery and timely initiation of breastfeeding are important measures for preventing prelacteal feeding in Raya Kobo district.

## Background

Breast milk is the ideal food [1] and superior to other forms of supplementary foods that an infant can receive [2]. It is a living fluid, containing all the necessary nutrients and hydration in the first six months of the child’s life. Breast milk is uncontaminated food that protects infants from infection and has an effect on the long term consequences, especially during adulthood, to prevent obesity and cardiovascular diseases [3,4]. Practice of giving pre-lacteal feeds was found also a key determinant of early cessation of full breastfeeding [5]. It is also associated with infant illness [6].

As global public health recommendations, international guidelines stress that infants should be exclusively breastfed for the first six months, then frequent and on demand breastfeeding should continue to 24 months [7,8]. Breastfeeding concern starts from the minute of birth. Evidences support that optimal breastfeeding practices rank among the most effective interventions to improve child health [9]. Optimal breastfeeding is associated with long term health and emotional benefits for the infant that reduces child morbidity and mortality [3,4,10].Suboptimal breastfeeding is responsible for 45% of neonatal infectious deaths, 30% of diarrheal deaths and 18% of acute respiratory deaths in children under 5 years [11]. It is estimated that optimal breastfeeding of children under two years of age has the potential to prevent 1.4 million deaths in children under five of age in the developing world annually [12]. In Ethiopia 18% of infant deaths could be attributed to poor breastfeeding practices [13]. Therefore to achieve child death related millennium development goals (MDGs), optimal breastfeeding is key and easiest intervention.

The most recent Ethiopian Demographic and Health Survey showed that nearly three children in every ten (27%) are given prelacteal feeds within the first three days of life. This practice is slightly more common in rural areas (27.5%) than in urban areas (24.2%) in Ethiopia. It is most commonly practiced in Somali Regional State (72.5%) followed by Amhara Regional State (47.8%) [4].

Avoidance of prelacteal feeding is one of the recommended practices to ensure optimal breastfeeding [14]. Prelacteal feeds are foods or drinks other than human breast milk that are given to newborns before breastfeeding initiation, usually on the first few days of life [2-4,15]. The term “makamesha” is used for the traditional prelacteal feeds in rural Amhara communities. It might include butter, cow’s milk, ersho (a traditional baking soda prepared by incubating the flour and double distilled water), water, honey, banana, herbal drinks and other substances [16]. Prelacteal feeding interferes with suckling, making breastfeeding more difficult to establish. Prelacteal feeding therefore remains a challenge to ensure optimal breastfeeding and adequate infant nutrition [1,3,17].

Prelacteal feeding is discouraged because it limits the infant’s frequency of suckling and exposes the baby to the risk of infection [4]. Infants who received prelacteal feeding were more likely to be stunted and wasted [18]. In West Gojjam, Ethiopia children who received prelacteal feeding were 1.8 times more likely to be stunted than children who were not subjected to prelacteal feeding [19]. Most Ethiopian women (89.5%) give birth at their home [4]. This may provide a supportive environment for infant feeding malpractice such as traditional prelacteal feeding.

Ethiopia has developed the National Infant and Young Child Feeding (IYCF) Guideline that discourages prelacteal feeding practices on newborns to achieve optimal breastfeeding [15]. Although prelacteal feeding is widely practiced in Ethiopia, the factors were not well studied in Ethiopia, particularly in Raya Kobo district. This study aimed to assess prelacteal feeding practices and associated factors among mothers of children aged less than 24 months in Raya Kobo district, North Eastern Ethiopia.

## Methods

### Study setting and participants

This study was conducted in Raya Kobo district of North Eastern Ethiopia from December 28, 2013 to January 18, 2014. Raya Kobo district is one of the thirteen districts of North Wello Zone, which is located 570 kms North-East of Addis Ababa. There are forty two kebeles (the smallest administrative units next to district in Ethiopia) under Raya Kobo district: five urban kebeles and thirty seven rural kebeles. In this district, there are forty two health posts and seven health centers. Each health post has two health extension workers [20].

A quantitative community based cross-sectional study was employed to survey mothers of children aged less than 24 months. The sample size was determined using a formula for estimation of single population proportion as follows:

$$ \mathrm{n}=\mathrm{D}\left[\frac{{\left(\mathrm{z}\frac{\upalpha}{2}\right)}^2\mathrm{p}\left(1-\mathrm{p}\right)}{{\mathrm{d}}^2}\right] $$

Where n = required sample size, Z = critical value for normal distribution at 95% confidence level (1.96), P = prevalence of prelacteal feeding in Amhara region (47.8%) [20], d = 0.05 (5% margin of error), D = 1.5 (design effect), and an estimated non-response rate of 10%.

### Sampling procedure

The sampling procedure was started from the stratification (assuming that the rural kebeles are relatively homogenous) of the thirty seven kebeles as rural and the five kebeles as urban (assuming that the urban kebeles are relatively homogenous). Out of the forty two kebeles, one urban kebele and seven rural kebeles were randomly selected using lottery method. Fourteen percent of the under-two population lives in urban areas and the remaining 86% lives in rural [20]. Therefore, based on population proportion, 544 mothers of children aged less than 24 months were taken from rural areas and 89 from urban areas.

Pre-survey was done before the actual day of data collection to know which households have the target mother-child pairs. As a result, there were 1,965 households having the targeted mother-child pairs in the selected eight kebeles (1,663 and 302 from rural and urban settings respectively).The total households divided by the sample size (the sampling fraction) was three. Then the first mother-child pair to be included in the sample was chosen randomly by picking one out of the first three houses, numbered one to three. At the time of survey, from each household unit one eligible mother who had a biological child aged less than 24 months was selected. Non-biological and mothers who are unable to communicate were excluded from the study.

During qualitative study, focus group discussions (FGD) and in-depth interviews were conducted to explore cultural beliefs about prelacteal feeding. The participants were selected purposively based on their role in the community. Two focus group discussions were undertaken in a group of grandmothers (woman who had at least one grandchild), each comprised of eight discussants. The in-depth interviews were undertaken with four traditional birth attendants (two trained and two untrained).

### Study variables

In this study, the outcome variable was prelacteal feeding practices among mothers of children aged less than 24 months. Prelacteal feeding was understood as providing foods and/or drinks other than human milk for the infant before the initiation of breastfeeding [3].The independent variables were maternal characteristics (age, educational status, religion, ethnicity), household characteristics (area of residence, household head and family size), husband educational status, child’s sex, antenatal care utilization, postnatal care utilization, place of delivery, mode of delivery, breastfeeding initiation and maternal knowledge on the risks associated with prelacteal feeding.

### Operational definitions

**Antenatal care utilization**: having at least one visit of health institution for checkup purpose during the pregnancy of the index child [21].

**Family size:** everybody living permanently in the same house was counted as family members.

**Postnatal care utilization**: receiving the care provided to the woman and the index child at least once during the six weeks period following delivery [22].

**Trained traditional birth attendant**: traditional birth attendant who receive training on mother-child cares during delivery.

**Untrained traditional birth attendant:** traditional birth attendant who can provide the delivery services without knowing the basic mother-child cares (did not take any training program).

### Data collection instrument, methods and process

Quantitative data were collected using a pre-tested, structured, interviewer-administered questionnaire adapted from Ethiopian Demographic and Health Survey [4] and the national nutrition survey questionnaire [23]. The adapted questionnaire was modified and contextualized to fit the local situation and the research objective. The EDHS definition of prelacteal feeding was “giving anything to drink other than breast milk in the first three days”. We operationalized prelacteal feeding eating or drinking something other than breast milk before initiation of breastfeeding. We assessed prelacteal feeding by this question “Before initiation of breastfeeding, was (NAME) given anything to drink and/or eat other than breast milk?”. We adapted the questionnaire to our study district culture for the type of prelacteal foods used. The questionnaire was prepared first in English, translated into Amharic, and then back into English by fluent speakers of both languages to check its consistency. The final Amharic version of the questionnaire was used to collect the data. The quantitative data were collected by eight high school graduates. The data collectors and the supervisors (two nurses having Bachelor of Science (BSc) were trained for three days (including practical work) by the principal investigators on the study instrument, consent form, how to interview and data collection procedures. Qualitative data were collected using focus group discussion and in-depth interview. Focus group discussions were facilitated by BSc nurse with the assistance of two nursing students (note takers). The in-depth interviews were also conducted by a female BSc nurse.

### Statistical analysis

The quantitative data were checked for completeness and inconsistencies. It was also cleaned, coded and entered into EpiData version 3.02, then exported to the SPSS 16.0 statistical package for analysis. Binary logistic regression analysis was performed; the crude odds ratio (COR) with 95% confidence interval was estimated to assess the association between each independent variable and the outcome variable, and to select candidate variables for the multivariate logistic regression analysis. Variables with p-value < 0.3 in the binary logistic regression analysis were considered in the multivariable logistic analysis. The Hosmer-Lemeshow goodness-of-fit with enter procedure was used to test for model fitness. Adjusted Odds Ratio (AOR) with 95% confidence interval was estimated to assess the strength of associations, and a p-value < 0.05 was used to declare the statistical significance in the final logistic model.

Qualitative data were transcribed in to an English text by the principal investigator (ML). Different ideas in the text were merged in their thematic areas and a thematic framework analysis was employed manually. The results were presented in narratives in triangulation with quantitative data.

### Ethical considerations

A letter of ethical approval was granted from the Institutional Health Research Ethics Review Committee (IHRERC) of Haramaya University. An official letter was written from School of Graduate Studies to Raya Kobo District Administration Office. Then permission and support letter was written to each selected Kebele. Informed written consent was taken from the participants before the interview. Illiterate mothers were consented by their thumb print after verbal consent. The participants were also assured about the confidentiality of the information they provided.

## Results

### Characteristics of sample

A total of 630 mother-child pairs were included in the study, resulting in a response rate of 99.5%. A total of 542(86%) of respondents were enrolled from rural settings while 14% were from urban. The mean (±SD) age of respondents was 27.91 years (±6.19) and ranged from 17 to 44 years. A majority (71.3%) of mothers were in the age group of 20–34 years. Only 9% of the respondents were household heads (single mothers). The mean (± SD) age of index children was 12.17 months (±6.72) (Table 1). Sixty three percent of respondents had attended at least one antenatal care (ANC) visit, 75.9% gave birth at home and 45.4% attended at least one postnatal care (PNC) visit after the birth of their index child (Table 2).

**Table 1:** **Socio-demographic characteristics of mothers of children aged less than 24 months in Raya Kobo district, North Eastern Ethiopia, January 2014**

**Variables**	**Frequency (n)**	**Percent (%)**
**Residence of mother**		
Rural	542	86.0
Urban	88	14.0
**Age of mother** (**in year**) (n = 630)		
< 20	47	7.5
20-34	449	71.3
> 34	134	21.2
**Maternal education status** (n = 630)		
No formal education	489	77.6
Primary and above	141	22.4
**Maternal marital status** (n = 630)		
Married	583	92.5
Divorced	30	4.8
Never married	17	2.7
**Religion of mother** (n = 630)		
Orthodox	423	67.1
Muslim	207	32.9
**Husband educational status** (n = 613)		
No formal education	515	84.0
Primary and above	98	16.0
**Age of the child (months**) (n = 630)		
< 6	129	20.5
6-11	161	25.6
12-17	159	25.2
18-23	181	28.7
**Family size** (n = 630)		
2	23	3.7
3-4	229	36.3
≥ 5	378	60.0

**Table 2:** **Distribution of respondents based on maternal and child health service utilization in Raya Kobo district, North Eastern Ethiopia, January 2014**

**MCH service utilization**	**Frequency (n)**	**Percent (%)**
**Antenatal care (ANC) visit** (n = 630)*		
Yes	396	62.9
No	234	37.1
**Number of ANC visits** (n = 396)		
1	38	9.6
2-3	274	69.2
≥ 4	84	21.2
**Counseling on breastfeeding at ANC** (n = 396)		
Yes	148	37.4
No	248	62.6
**Place of delivery** (n = 630)		
Home	478	75.9
Health institution	152	24.1
**Delivery attendant** (n = 630)		
Untrained traditional birth attendant	271	43.0
No one	198	31.4
Health professional	152	24.2
Trained traditional birth attendant	9	1.4
**Mode of delivery** (n = 630)		
Vaginal delivery	616	97.8
Caesarean section	14	2.2
**Postnatal care (PNC) visit** (n = 630)*		
Yes	286	45.4
No	344	54.6
**Counseling on breastfeeding at PNC** (n = 286)		
Yes	121	42.3
No	165	57.7

*at least one visit.

### Feeding practices

Almost all respondents (99%) had “ever” breastfed their index child. Of those who had ever breastfed, 447 (71.8%) mothers initiated breastfeeding within one hour of birth. Exclusive breastfeeding under six months was practiced by 96.1% of mothers. Continued breastfeeding at one year (children of 12–15 months of age) was 120 (99.2%). Continued breastfeeding at two years (20–23 months of age.) was 110 (96.5%).

Of the 623 mothers who had ever breastfed their index child, 242 (38.8%; 95% CI: 35.0%, 43.0%) reported giving prelacteal feeds to their children. The most common prelacteal foods were sugar solution (38%) and raw butter (32%).Three hundred and ninety (62.6%) of respondents who had ever breastfed did not know of the risks associated with prelacteal feeding. Of the respondents who had practiced prelacteal feeding, 73%mothers did not report any purported advantages of prelacteal feeding for infants (Table 3).

**Table 3:** **Prelacteal feeding practices of respondents who had ever breastfed their index child in Raya Kobo district, North Eastern Ethiopia, January 2014**

**Variable**	**Frequency (n)**	**Percent (%)**
**Prelacteal feeding practice for the index child **(n = 623)		
Yes	242	38.8
No	381	61.2
**Prelacteal foods **(n = 242)*		
Sugar solution	92	38.0
Butter	78	32.2
Ersho^$^	31	12.8
Honey	30	12.4
Plain water	9	3.7
Milk other than breast milk	2	0.8
**Purported prelacteal feeding advantage **(n = 242)^†^		
Do not know	177	73.1
Behavioral modification	28	11.6
For child health	24	9.9
For child growth	13	5.4
**Knowing risks associated with prelacteal feeding **(n = 623)*		
Do not know	390	62.6
Diarrhea	213	34.2
Infection	26	4.2
Vomiting	10	1.6
Poor growth	10	1.6

*Variables that have multiple responses

^†^There are no recognized benefits associated with prelacteal feeding. The medical community defines all prelacteal feeding as (potentially) dangerous [3,4].

^$^Ersho is a traditional baking soda prepared by incubating the flour and double distilled water.

Forty four (18.2%) mothers were influenced by other individuals to practice prelacteal feeding. The influential individuals were husbands (47.7%), untrained traditional birth attendants (38.7%) and grandmothers of the index child (13.6%). The other reasons were: to keep baby’s mouth moist (17.8%), to keep baby’s body warm (3.7%), mother was sick (3.7%), delayed lactation (3.3%), insufficient breast milk (2.0%) and 1.2% were due to inability of the baby to suck breast milk.

Prelacteal food and prelacteal feeding was defined by a 40 years old mother as *“Prelacteal food is a food that is given for infants before breastfeeding initiation; in this society it is called “makamesha” and the practice is called “makames” in Amharic” (FGD- Grandmother)*.

In relation to purported benefits *of prelacteal feeding, a 48 years old mother said, “we provide raw butter “lega-kibe” for infants after the expulsion of the placenta for two basic reasons: it cleans their stomach and it helps to guide the infants’ future behavior” (FGD- Grandmother). A 37 years old woman said, “even though it is not practiced today, five years ago raw butter was given to clean infant’s stomach”(In-depth interview-Trained Traditional Birth Attendant)*.

### Factors associated with prelacteal feeding

The binary logistic regression analysis showed that living in rural places, giving birth at home, initiating breastfeeding after one hour of delivery and mothers who did not know the risks associated with prelacteal feeding were statistically associated with prelacteal feeding.

In multivariable logistic regression analysis giving birth at home, initiating breastfeeding after one hour of delivery and mothers who did not know the risks associated with prelacteal feeding remained as statistically significant positive predictors of prelacteal feeding practice. Those mothers who gave birth at home were seven times more likely to practice prelacteal feeding as compared to mothers who gave birth at health institution (AOR:7.10;95% CI (3.91,12.98)). Mothers who initiated breastfeeding after one hour of delivery were nearly three times (AOR: 2.70; 95% CI (1.78, 3.99)) more likely to practice prelacteal feeding compared to mothers who initiated breastfeeding within one hour of delivery. Mothers who did not know the risks associated with prelacteal feeding were 3.7 times (AOR: 3.70; 95% CI: 2.44, 5.53) more likely to practice prelacteal feeding than their counterparts (Table 4).

**Table 4:** **Factors associated with prelacteal feeding practices among mothers of children aged less than 24 months in Raya kobo district, January 2014**

**Variable**	**Prelacteal feeding n (%)**	**Crude odds ratio (95%CI)**	**Adjusted odds ratio (95%CI)**
**Residence**			
Urban	21 (23.9)	1	1
Rural	221 (41.3)	2.3 (1.34, 3.78)*	1.5 (0.80, 2.76)
**Husband’s educational status**			
No formal education	205 (40.3)	1.5 (0.95, 2.39)	0.7 (0.41, 1.28)
Primary and above	30 (30.9)	1	1
**Antenatal care visit** ^**†**^			
Yes	144 (36.9)	1	1
No	98 (42.1)	1.2 (0.89, 1.73)	0.7 (0.47, 1.01)
**Place of delivery**			
Home	224 (47.5)	6.7 (3.95, 11.27)*	7.1 (3.91, 12.98)*
Health institution	18 (11.9)	1	1
**Mode of delivery**			
Cesarean section delivery	3 (21.4)	1	1
Vaginal delivery	239 (39.2)	2.4 (0.65, 8.58)	1.0 (0.24, 4.14)
**Breastfeeding initiation time**			
≤ 1 hour	142 (31.8)	1	1
> 1 hour	100 (56.8)	2.8 (1.97, 4.04)*	2.7 (1.78, 3.99)*
**Mother knows risks associated with prelacteal feeding**			
Yes	51 (21.8)	1	1
No	191 (49.0)	3.4 (2.37, 4.95)*	3.7 (2.44, 5.53)*

*Statistically significant variables at p <0.05, CI = Confidence Interval.^†^at least one visit. Hosmer-Lemeshow goodness-of-fit = 0.837.

*In support of this idea, a 40 years old mother said, “in our community infant without prelacteal feeding is not acceptable because infant may die unless prelacteal feeding is provided” (FGD- Grandmother).*

*A 43 years old mother said, “Every infant should be provided with food before starting breastfeeding. That protects infants from the negative effects of the “evil eyes” of other people.” (FGD- Grandmother).*

*A 45 years old untrained traditional birth attendant (UTTBA) said, “prelacteal foods can prevent infants from disease, therefore I recommend all mothers to give these foods to their infants immediately after birth” (In-depth interview-UTTBA).*

## Discussion

The prevalence of prelacteal feeding in Raya Kobo district was about 39%. Giving birth at home, initiating breastfeeding after one hour of delivery and mothers who did not know the risks associated with prelacteal feeding were positive predictors of prelacteal feeding practice.

Breastfeeding is nearly universal in Raya Kobo district. However, breastfeeding practices are sub-optimal in the district due to the wide spread introduction of prelacteal feeds. This study found the prevalence of prelacteal feeding in Raya Kobo (38.8%) to be higher than the prevalence of prelacteal feeding (27%)in the city of Bahir Dar [24], which is in the same region and is inhabited by the same ethnic group. This could be due to the difference in the study setting on which Bahir Dar study was conducted in urban setting only. Prelacteal feeding practice was more prevalent in the Raya Kobo district as compared to all regional prevalence’s of Ethiopia except Amhara and Somali [4].A relatively similar prevalence of prelacteal feeding (45.4%) was reported from Harari Region Public Health Facilities, Eastern Ethiopia [25] and in a study from Western Uganda (31.3%) [26]. The highest prevalences of prelacteal feeding were reported from Kersa district of Ethiopia (75.8%) [27] and Vietnam (73.3%) [28].

This study showed that mothers who gave birth with the help of traditional birth attendants (55%) were more likely to practice prelacteal feeding compared to mothers assisted by trained health professionals (13%). Similarly, from previous survey mothers assisted by traditional birth attendants (34%) were more likely to give prelacteal feeds to their children than mothers assisted by trained health professionals (21%) in Ethiopia [4]. This may be due to the influence of traditional birth attendants or family members and friends on mothers of children to practice prelacteal feeding at home. Alternatively, mothers who gave birth at health institution may be encouraged by health professionals to avoid prelacteal feeding.

Mothers who did not know the risks associated with prelacteal feeding were 3.7 times more likely to practice prelacteal feeding compared to mothers who knew the risks associated with prelacteal feeding. This study parallels a study from Vietnam that reported odds of feeding prelacteal declined with increased breastfeeding knowledge [28]. Focus group discussion findings in the study area showed that, mothers were feeding their infants with raw butter “*lega-kibe*” because of the mistaken/inaccurate belief that prelacteal feeding cleans infant’s stomach. Furthermore, discussants in the study area stated that it is a very common belief that a child takes the behavior of the person who gives prelacteal feeding. Similarly, in Jimma Arjo district mothers gave rue (“tena-addam”) and butter to infants to protect them against the common cold and stomach ache [29].

Late initiation of breastfeeding was associated with prelacteal feeding practice. Compared to mothers who initiated breastfeeding within one hour of delivery, mothers who initiated breastfeeding after one hour of delivery were 2.7 times more likely to practice prelacteal feeding. This is consistent with studies from Eastern Ethiopia and Western Uganda [25,26]. This might be explained as the time interval between delivery and breastfeeding initiation increases, there will be more time for infant feeding malpractices like prelacteal feeding. In fact the prelacteal feeding might be also the reason for late initiation of breastfeeding.

Mothers who delivered the index child at home were seven times more likely to practice prelacteal feeding compared to mothers who gave birth at health institution. This is consistent with a study from eastern Ethiopia [25]. Giving birth at home create a favorable environment for different socio-cultural factors, like family influence and birth attendants that can influence mothers to practice prelacteal feeding. In addition, mothers who gave birth in health institution might be advised by health professionals about the risks associated with prelacteal feeding.

In this study mothers educational status was not significantly associated with avoidance of prelacteal feeding. This is consistent with the study from India [30]. However a contrary finding were also reported [31]. Mothers’ education affects proper child feeding practices through enhancing their knowledge from different information sources like newspapers by independent learning. But in this study area most of the mothers were from rural areas and do not have access for newspaper regarding prelacteal feeding. Though the mothers have better educational status, it might not be useful for increasing their knowledge of prelacteal feeding and their practices. The other possible justification is that even though mothers are aware of prelacteal feeding, they might be influenced by the local community members to use prelacteal feeding. So that having better educational status or not might not affect prelacteal feeding practices. This implies that, although all women do need formal education, nutrition education is a short-term intervention that will have a considerable impact on the community.

Findings from this study have substantial contribution in the promotion of optimal breastfeeding practices and the achievement of the millennium development goal in reducing child mortality in Ethiopia. The strength of this study was it included both quantitative and qualitative methods. However, the limitation of this study was that information obtained from mothers having children aged less than 24 months is subject to recall bias. The study also shares the limitation of the cross-sectional study design.

## Conclusion

Prelacteal feeding is commonly practiced in Raya Kobo district. This makes breastfeeding practices sub-optimal in the district. Home delivery, improper commencement of breastfeeding after birth and poor maternal knowledge about the risks associated with prelacteal feeding are important positive predictors of prelacteal feeding practice. Awareness of the risks associated with prelacteal feeding, promotion of institutional delivery and timely initiation of breastfeeding are recommended interventions to reduce prelacteal feeding practices in Raya Kobo district. Interventions to reduce prelacteal feeding should also target traditional birth attendants within the study area.
